# Surgical Removal of Circumferentially Leaked Polymethyl Methacrylate in the Epidural Space of the Thoracic Spine after Percutaneous Vertebroplasty

**DOI:** 10.1055/s-0037-1598030

**Published:** 2017-02-10

**Authors:** Kenichiro Kita, Yoichiro Takata, Kosaku Higashino, Kazuta Yamashita, Fumitake Tezuka, Toshinori Sakai, Akihiro Nagamachi, Koichi Sairyo

**Affiliations:** 1 Department of Orthopedics, Tokushima University, Tokushima, Japan

**Keywords:** percutaneous vertebroplasty, cement leakage, neurologic deficit, surgical removal

## Abstract

**Background** The major complication of percutaneous vertebroplasty (PVP) using polymethyl methacrylate (PMMA) is epidural leakage of PMMA that damages the spinal cord.

**Methods** This is a case report.

**Result** A 77-year-old man presented to our institution with a 6-month history of muscle weakness and an intolerable burning sensation of both lower limbs after PVP with PMMA for thoracic compression fracture at T7 at another hospital. His past medical history was significant for hypertension. He had no history of smoking and alcohol. Computed tomography revealed massive leakage of PMMA into the T6 and T7 spinal canal circumferentially surrounding the spinal cord that caused marked encroachment of the thecal sac. Magnetic resonance images revealed cord compression and intramedullary signal change from T6 to T7 level. After we verified that the leaked PMMA could be easily detached from the dura mater in the cadaveric lumbar spine, surgical decompression and removal of epidural PMMA was performed. The leaked PMMA was carefully thinned down with a high-speed diamond burr. Eight pieces of PMMA were detached from the dura mater easily without causing a dural tear. No neurologic deterioration was observed in the postoperative period. The burning sensation resolved, but the muscle weakness remained unchanged. One and a half years postoperatively, the muscle weakness has improved to ⅘ on the manual muscle strength test, but he could not walk without an aid because of spasticity.

**Conclusion** This report demonstrates the catastrophic epidural extrusion of PMMA following PVP. Extravasated PMMA can be removed through a working space created by means of laminectomy and subtraction of the affected pedicle. Spine surgeons should recognize the possible neurologic complications of PVP and be prepared to treat them using suitable approaches.

Osteoporotic vertebral fractures result in significant mortality and morbidity with prolonged and intractable pain. The technique of percutaneous vertebroplasty (PVP) was first reported in 1987 as a treatment for vertebral hemangiomas. 1 PVP with polymethyl methacrylate (PMMA) is a minimally invasive procedure to stabilize a vertebral fracture and is widely used for their treatment. However, several complications, such as leakage of PMMA, adjacent fracture, pulmonary embolism, and systemic toxicity of the monomer, were reported. One of the severe complications of PVP with PMMA is epidural leakage of PMMA that may cause a neurologic deficit.

We report a case of epidural cement leakage following PVP with PMMA causing neurologic damage, which was surgically removed successfully.

## Case Report

**Fig. 1:**
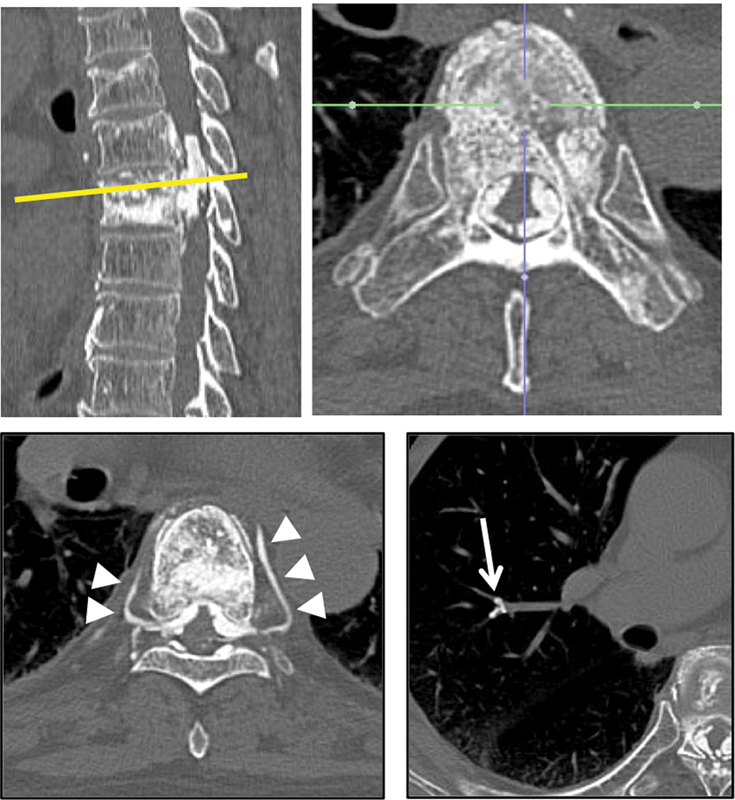
Upper left panel: preoperative computed tomography (CT) sagittal image showing leaked polymethyl methacrylate (PMMA) into spinal canal from T6 thorough T7. Upper right panel: preoperative CT axial image showing circumferentially leaked PMMA surrounding the thecal sac. Lower left panel: axial CT image showing leaked PMMA into the segmental vein bilaterally (white arrow head). Lower right panel: axial CT image showing leaked PMMA into right pulmonary artery (white arrow).

**Fig. 2:**
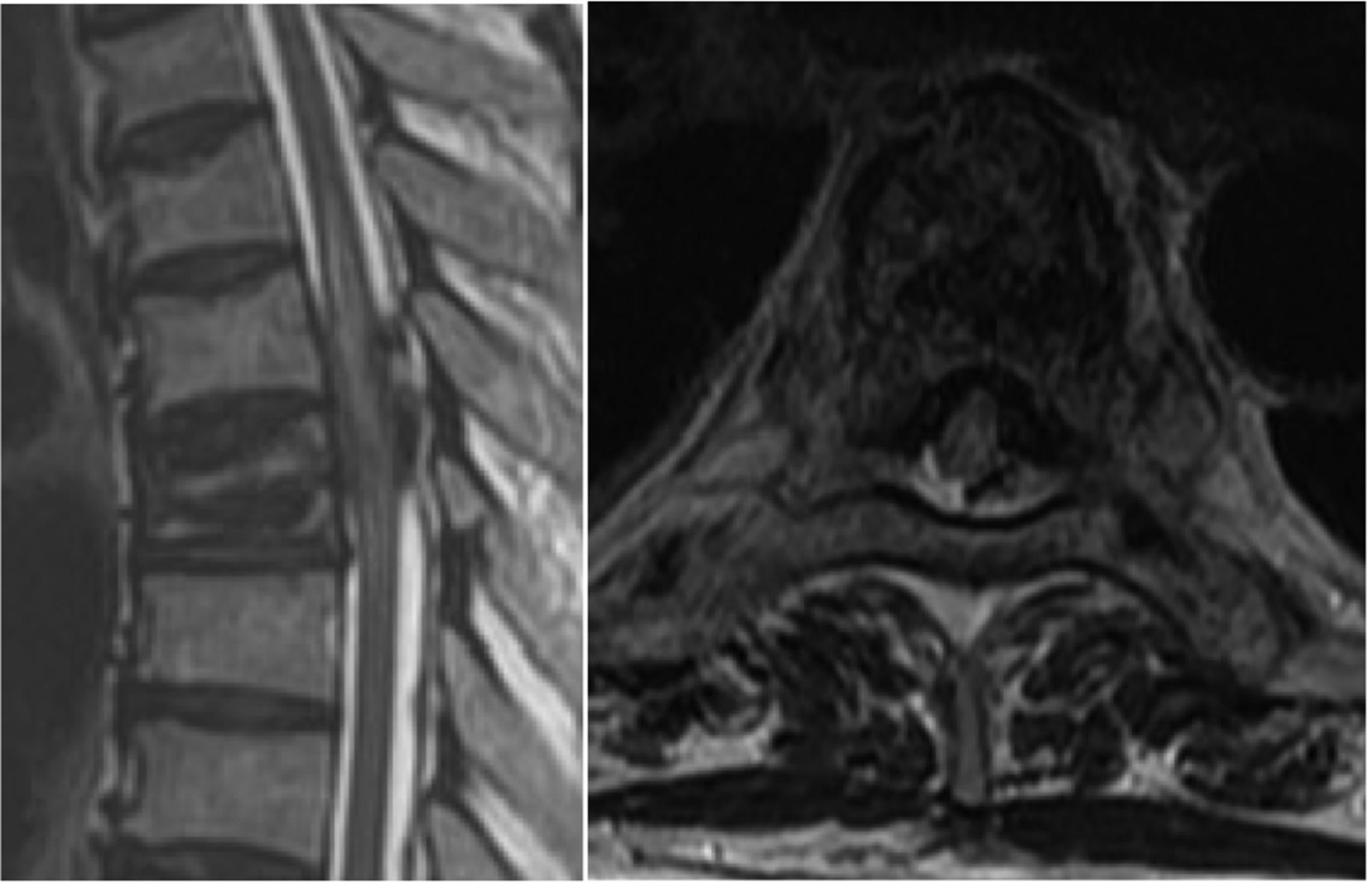
Magnetic resonance sagittal and axial images showing compressed spinal cord by leaked polymethyl methacrylate and intramedullary high-signal change.

A 77-year-old man presented to our institution with a 6-month history of muscle weakness and an intolerable burning sensation of both lower limbs after PVP with PMMA for thoracic compression fracture at T7. He underwent PVP with PMMA just 2 weeks after injury. His past medical history was significant for hypertension. He had no history of smoking and alcohol. Computed tomography (CT) revealed a massive leakage of PMMA into the T6 and T7 spinal canal circumferentially surrounding the spinal cord that caused marked encroachment of the thecal sac, and small pieces of leaked PMMA embolized to the left pulmonary artery ( Fig. 1 ). CT images showed that PMMA injection through the unilateral pedicle approach was used. Magnetic resonance imaging (MRI) revealed cord compression due to epidural PMMA and intramedullary signal change from T6 to T7 level ( Fig. 2 ). Neurologic examination revealed that the muscle strength of both lower limbs were decreased to the 2 to 3 level in the manual muscle strength test: iliopsoas (2 to ⅗), quadriceps (⅘), tibialis anterior (2 to ⅗), extensor hallucis longus (2 to ⅗), and flexor hallucis longus (⅘). Sensory disturbance including touch and cold sensation existed below T7 level. Babinski reflex was positive on the left side. Patella tendon and Achilles tendon reflex were normoactive on both sides. Bowel and bladder function remained normal.

**Fig. 3:**
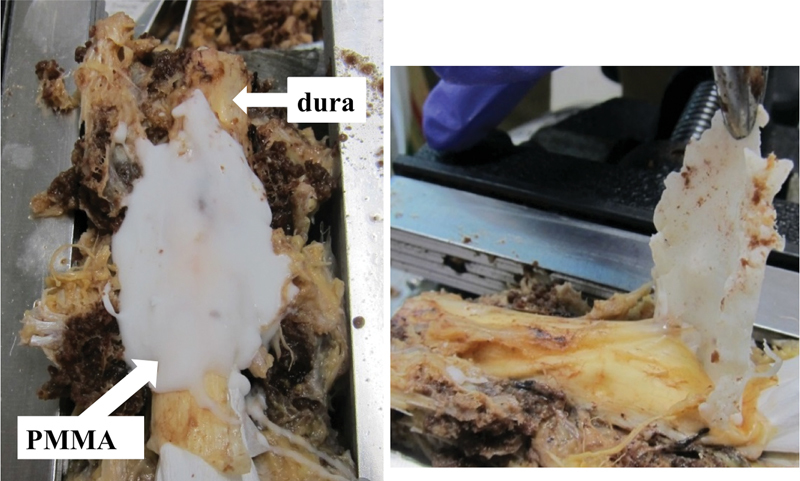
Cadaveric study to evaluate the safety of removal of polymethyl methacrylate (PMMA) from dura mater. Left panel: PMMA was plastered on the dura mater to reproduce PMMA leakage into spinal canal. Right panel: PMMA was easily detached from dura mater of the cadaveric spine.

To verify the safety of removal of PMMA from the dura mater, we conducted a cadaver study. The dura of formalin-fixed cadaveric lumbar spine was exposed. PMMA was plastered on the dura to reproduce the epidural leakage ( Fig. 3 ). The adhesion of PMMA cement and dura was mild. PMMA was easily detached from the dura.

**Fig. 4:**
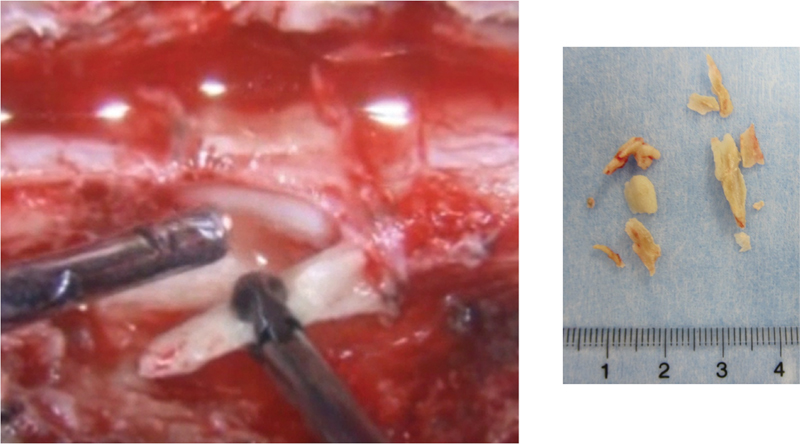
Left: intraoperative image showing polymethyl methacrylate (PMMA) detached from the dura mater. Right: eight pieces of detached PMMA fragments.

Surgical decompression and removal of epidural PMMA were performed. After temporary fixation with posterior instrumentation from T5 to T9, laminectomy of T6–T7 and bilateral facetectomies at T6–T7, and bilateral pedicle subtraction of T7 were performed. The extravasated mass of PMMA was carefully thinned down with a high-speed diamond burr. Eight pieces of PMMA were detached from the dura mater relatively easily without causing a dural tear ( Fig. 4 ). No neurologic deterioration was observed in the postoperative period. The burning sensation resolved but muscle weakness remained unchanged. MRI revealed an intramedullary high signal change at the level of T6–T7. One year postoperatively, the muscle weakness had improved to ⅘ level on manual muscle strength testing, but he cannot ambulate without an aid because of spasticity.

## Discussion

The incidence of PMMA leakage after vertebroplasty is reported to be 22 to 38%. 2 3 4 5 Yeom et al classified cement leakage into three types: type B is through the basivertebral vein, type S is through the segmental vein, and type C is through a cortical defect. The percentages of type B, type S, and type C are 38, 39, and 23%, respectively. 5 We speculate that both type B and type S were occurred in this case ( Fig. 1 ). In type B, leaked cement was distributed symmetrically into the spinal canal. In type S, PMMA leaked into the segmental vein bilaterally. Despite the high rate of PMMA leakage into the spinal canal, extrusions of PMMA cement are usually clinically asymptomatic. But epidural leakage of cement may cause a neurologic deficit because of the direct mass effect, and thermal injury to the spinal cord or nerve root. In this case, leaked PMMA was massive and led to catastrophic neurologic damage.

Many factors contributing to the occurrence of intracanal leakage of PMMA were reported, such as viscosity of PMMA, 6 amount of PMMA injected, 7 bilateral or unilateral pedicular approach, 3 7 period of PVP after injury, 8 adequate intraoperative radiograph, 9 and preoperative verification of the fracture pattern (posterior wall or pedicle fracture). 10 In this case, viscosity of injected PMMA must have been low, and amount of injected PMMA must have been too much because the leaked PMMA was spread around the spinal canal and spread into the caudal vertebral level and into the segmental vein and pulmonary artery. In this case, unilateral pedicle approach was used. Unilateral transpedicular approach increases the risk of excessive local pressure during injection, leading to cement leakage. The period of PVP after injury was just 2 weeks in this case. It might be relatively early because fractured vertebra was still unstable at 2 weeks after injury. In this case, we inferred that operating surgeon failed to pay enough attention to cement leakage.

If symptomatic cement leakage occurred, surgical treatment will be needed. Immediate surgical decompression with removal of bone cement should be helpful in case of epidural leak to prevent new-onset neurologic deficits if their cause is a direct mass effect. It has been reported that when complete paralysis developed, no neurologic recovery was obtained even after emergent decompression surgery. 6 11 12 It has also been reported that patients with incomplete paraplegia who were treated on the same day or the day after PVP showed good recovery. 4 12 13 Therefore, prompt diagnosis and treatment are important.

Cement leakage should be suspected in patients with an abrupt onset of uninterpretable symptoms after vertebroplasty. 14 Advanced imaging studies are necessary for confirmation of the diagnosis and evaluation of severity. In this case, cement leakage seemed to be type B, but PMMA leaked circumferentially into the spinal canal. The reason is that the low-viscosity PMMA cement might have been injected with a high pressure through the unilateral pedicular approach with an inappropriate technique and lack of attention. Intermittent injection without a continuous forceful squeeze is necessary to handle the volume and rate of cement injection. 14 In this case, the patient complained of neurologic symptoms immediately after PVP, but he had not undergone any treatment for 6 months. When he came to our department, the neurologic deficit had already progressed and recovery was poor even after decompression surgery. Surgeons must keep in mind that leakage of cement is not a rare complication of PVP and that a neurologic deficit after PVP may suggest cement leakage into the spinal canal. PVP should be done in a medical institution where surgical treatment for neurologic complication can be performed with sufficient knowledge and technique.

## Conclusion

We report a case of paraplegia resulting from spinal cord compression by circumferentially leaked PMMA cement after PVP. The cadaveric study revealed that adhesion between PMMA and dura was mild and easy to detach. Intraoperatively, leaked PMMA was successfully removed by a high-speed diamond burr. Immediate treatment for a neurologic deficit due to PMMA leakage is needed. Spine surgeons should recognize the possible neurologic complications of PVP and be prepared to treat it using suitable approaches.
